# Active Neurodynamics at Home in Patients with Knee Osteoarthritis: A Feasibility Study

**DOI:** 10.3390/jcm12206635

**Published:** 2023-10-20

**Authors:** Beatriz Serrano-García, Francisco Forriol-Campos, Juan Carlos Zuil-Escobar

**Affiliations:** 1 Escuela Internacional de Doctorado (CEINDO), Universidad San Pablo-CEU, CEU Universities, Urbanización Montepríncipe, 28660 Boadilla del Monte, Spain; 2 Hospital Ruber Juan Bravo, Calle Maldonado 52, 28006 Madrid, Spain; 3 Departamento de Ciencias Médicas Clínicas, Facultad de Medicina, Universidad San Pablo-CEU, CEU Universities, Urbanización Montepríncipe, 28660 Boadilla del Monte, Spain; fforriol@ceu.es; 4 Departamento de Fisioterapia, Facultad de Medicina, Universidad San Pablo-CEU, CEU Universities, Urbanización Montepríncipe, 28660 Boadilla del Monte, Spain; jczuil@ceu.es

**Keywords:** feasibility studies, physical therapy modalities, osteoarthritis, knee, pain, quality of life, self care

## Abstract

The aim was to evaluate the feasibility of a home-based neurodynamic programme for patients with knee osteoarthritis (KO). Thirty participants (70% women) ≥ 50 years old with KO (Kellgren–Lawrence grades I–II) were included. Active mobilisation of the femoral nerve was performed at home over a period of 6–8 weeks. The feasibility of the programme was assessed using a survey that included questions related to understanding of the activity; adherence to the intervention; the burden caused by the intervention; self-perceived effects on the participant; follow-up; the barriers; and facilitators. Pain intensity, using the numerical rating scale (NRS); pressure pain thresholds (PPT); temporal assessment; pain modulation; Knee Injury and Osteoarthritis Outcome Score (KOOS), 12-item Short Form Survey questionnaire (SF-12), and the Central Sensitization Inventory questionnaire (CSI) were also collected, before and after the intervention. All patients performed the intervention, completed at least 42 days of activity, and considered the exercise adequate, with 28 participants (93.3%) reporting that the intervention was good for them. Statistically significant values (*p* < 0.05) were found for NRS, elbow PPT, external knee PPT, internal knee PPT, elbow CPM, CSI, and KOOS. Home-based active neurodynamic treatment has been shown to be a feasible and safe intervention for KO patients. In addition, this intervention has shown positive effects on pain and function.

## 1. Introduction

The prevalence of knee osteoarthritis (KO) has doubled since the mid-20th century and is expected to continue to increase in the future [1]. The knee is the joint most affected by osteoarthritis [2] and 250 million people worldwide suffer from KO [3]. Age is a risk factor for KO, which causes dysfunction and pain in the elderly [1,4,5]. Its economic burden is estimated at 89,100 dollars per person per year [2,3].

Symptoms of KO include pain, muscle weakness, stiffness, dysfunction, and reduced quality of life [6,7]. Pain is a multifactorial phenomenon involving structural, psychosocial and neurophysiological factors [8]. In KO, inflammatory mediators have been found in somatic structures, altering afferent inputs and causing changes in the nervous system, which can lead to central sensitization [9].

The goal of KO treatment is to reduce its symptoms and to improve the quality of life [10] and therefore long-term interventions are needed [11]. Conservative treatment includes exercise and manual therapy [12]. These treatments require frequent and regular visits to specialised centres, which can be challenging as many patients are elderly. Furthermore, they are expensive, require transport and are time-consuming [13].

Neurodynamics is the gliding of nerves by mobilizing and positioning various joints to decrease nerve tension [14]. It has been suggested that increased gliding between the nerve and associated tissues, mobilises intraneural fluid and increases nerve mobility, leading to a potential reduction in symptoms [15]. In addition, Shacklock et al. [16] pointed out that neurodynamics can lead to improvements in nerve conduction and blood flow. Neurodynamics has previously been shown to increase knee range of motion and decrease pain. Herrington et al. [17] found a statistically significant increase in knee range of motion in healthy women using neurodynamics, and Lau et al. [18] reported that neurodynamics was beneficial for pain and self-efficacy in patients with rheumatoid arthritis. However, the effect of neural mobilisation on people suffering from KO has not been previously studied.

Home-based programmes can play an important role in reducing time and costs [19]. Previous studies have shown that different home-based programmes (including exercise, health education or neuromuscular electrical stimulation) reduce the symptoms and increase functional ability in people with KO [20,21,22], and these programmes have shown similar results to office-based programmes [13,22].

The success of home-based rehabilitation programmes depends on the feasibility of the interventions and on patient adherence [23]. A previous study in which participants completed a home-based muscle strengthening and joint flexibility programme found high adherence rates (96–100%) [24]. Adherence can be increased by using strategies such as sending text reminders [25] or explanatory leaflets [26]. In addition, home-based rehabilitation programmes are more effective when led by physiotherapists [27].

As mentioned above, neurodynamics have shown positive effects in patients with rheumatoid arthritis [18], but there is no previous research on its effects in patients with KO. The aim of this study is to analyse the feasibility of a home-based neurodynamic programme for patients with KO and to assess the feasibility of outcome measures for a future clinical study. Therefore, this study aims to combine the potential benefits of neurodynamic treatments for patients with KO with the economic and time advantages of home-based rehabilitation programmes.

## 2. Materials and Methods

### 2.1. Design

This research is part of a main study to evaluate the effects of a home-based neurodynamic programme aimed at KO patients (NCT05375448).

The research was approved by the Ethics Committee of Fundación Jimenez Diaz Hospital (EO222-20_HRJB).

### 2.2. Participants

Inclusion criteria were: patients ≥ 50 years old, with knee pain, diagnosed with KO according to the American College of Rheumatology’s criteria [28], and grade I or II on the Kellgren–Lawrence radiographic scale [29]. Exclusion criteria were: suffering from any disease that could cause pain in the lower limbs; suffering from chronic diseases that could be considered as perpetuating factors (e.g., fibromyalgia); corticosteroids or local anaesthetics infiltration during the year before the start of the study or during the follow-up period; painkiller intake 24 h before assessments; consumption of substances that could interfere with the treatment; contraindications to mobilisation or exercise; previous diagnosis of myopathy or neuropathy (lumbosacral plexus); and cognitive deficits (Alzheimer’s, dementia). Participants were informed of the aims and methodology of the research and each signed an informed consent form. The main study was approved by the Ethics Committee of Fundación Jimenez Diaz Hospital.

### 2.3. Intervention

**Figure 1:**
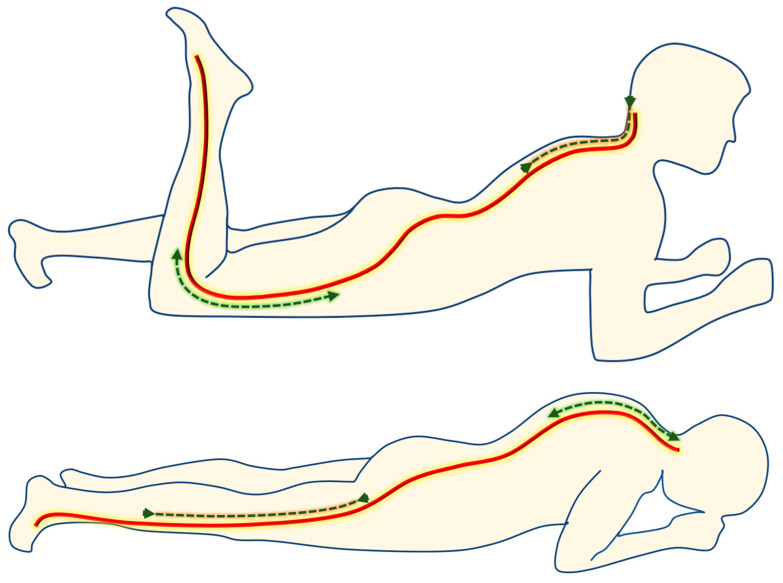
Mobilisation of the femoral nerve.

Participants were instructed by a physiotherapist to perform active femoral nerve mobilisation using the following method: while in the prone position, supported on the forearms with a slight spinal extension, knee flexion and cervical spine extension were performed, followed by the opposite movements [30] (Figure 1). Patients were provided with a video to demonstrate the movements and asked to perform the exercise for 6–8 weeks (20 repetitions/day: 10 repetitions in the morning and 10 repetitions in the evening). Treatment was followed up weekly by telephone and, in case of doubt, the physiotherapist supervised a session on site. Daily repetitions were also self-reported.

### 2.4. Variables

The variables of age, gender, height, weight, body mass index (BMI), Kellgren–Lawrence radiographic scale, lower limb deformities/dissymmetry, and whether participants were taking analgesics were collected at baseline.

Feasibility was assessed using a post-intervention survey including questions about understanding of the activity, adherence to the intervention, burden, subjective effect, follow-up, barriers, and facilitators (Supplementary S1).

To assess the feasibility of data collection, outcome measures of the main study were also recorded. Data were collected twice: at the baseline (T0) and after the intervention (T1). The variables included were:

Pain intensity, which was assessed using the Numerical Rating Scale (NRS), ranging from 0 (“no pain”) to 10 (“the most intense pain imaginable”) [31]. The NRS has shown acceptable reliability in elderly people and also correlates strongly with other pain scales [31].

Pressure pain threshold (PPT) was measured with an analogue algometer (WAGNER). The analogue algometer (1 cm^2^/surface) was placed perpendicular to the skin and the pressure was increased by 1 kg/cm^2^/s until the first painful sensation. Three measurements were recorded at 30 s intervals. Measurements were taken at three points: two points on the patella area (3 cm medial to the medial border and 3 cm lateral to the lateral border), and one point on the ipsilateral radial extensor carpi radialis longus (5 cm from to the lateral epicondyle).

Temporal summation (TS) and conditioned pain modulation (CPM) were evaluated at the points indicated above. For TS, a sequence of 10 pulses was applied at each point, with a pressure increase of 2 kg/s, held for 1 s at each pulse. Pain intensity at the 1st, 5th, and 10th pulse was assessed using a NRS.

CPM was assessed after a five-minute rest. A sphygmomanometer was placed on the contralateral arm. It was inflated at a rate of 20 mm Hg/second until the first painful sensation and held for 30 s. The temporal assessment was then repeated with the cuff held at a pressure that produced a painful intensity of 3/10 on the NRS [32].

The Spanish Central Sensitization Inventory (CSI) questionnaire, which is validated and shows high reliability, was used to identify symptoms related to central sensitization [32]. The score was interpreted as follows: subclinical = 0–29; mild = 30–39; moderate = 40–49; severe = 50–59; and extreme = 60–100.

The Spanish version of the 12-item Short Form Survey (SF-12) questionnaire [33] was used to collect information on capacity, well-being, and physical function.

The Knee Injury and Osteoarthritis Outcome Score (KOOS) questionnaire consists of 42 items, and presents 5 subscales which are scored individually: pain (KOOSP) [9 items], symptoms (KOOSS) [7 items], activities of daily living (KOOSADL) [17 items], functioning in sports and recreation (KOOSSR) [5 items], and quality of life (KOOSQL) [4 items]. Each item presents 5 options (none, a little, moderate, severe, and extreme). For the final score, all items are summed, and this score is converted to a scale from 0 to 100, where 0 represents the most extreme problems and 100 represents no knee problems [34]. The Spanish version is validated and reliable in patients with KO [34].

### 2.5. Statistical Analysis

Data analysis was carried out using the SPSS statistical programme (v.24, IBM, SPSS Inc., Chicago, IL, USA). The Kolmogorov–Smirnov test was used to assess the normal distribution of quantitative variables. A descriptive study was performed, using means, standard deviations, medians, and interquartile ranges for quantitative variables, and frequencies and percentages for qualitative data. Student’s *t*-test or the Wilcoxon test were used to compare variables at T0 and T1. Student’s *t*-test was only carried out when the variables showed a normal distribution at both T0 and T1, and the Wilcoxon test was used in the opposite situations. For variables that showed a normal distribution, the effect size was assessed by Cohen’s d [35], interpreted as follows: small effect (d = 0.2), medium effect (d = 0.5), and large effect (d = 0.8). For variables that did not show a normal distribution, the effect size was calculated using r of the Wilcoxon test [36,37], interpreted as small effect (r = 0.1), medium effect (r = 0.3), and large effect (r = 0.5) [36].

## 3. Results

### 3.1. Participants

Thirty participants (66.27 ± 8.93 years old) with KO took part in the research. Most of the participants were women (70%), with grade II in the Kellgren–Lawrence scale (80%), without lower limb deformities (93.33%), and not taking analgesics (83.33%). Table 1 shows the demographic variables. All the demographic variables showed a normal distribution in the Kolmogorov–Smirnov test (*p* < 0.05).

### 3.2. Recruitment

**Figure 2:**
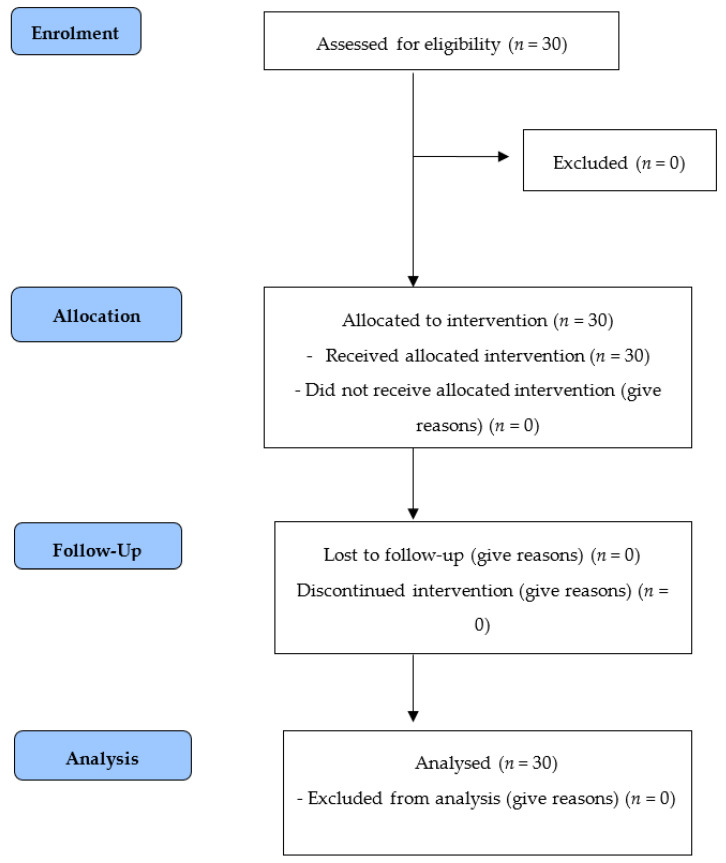
Flow chart of participant selection according to CONSORT.

Thirty participants met the inclusion criteria. All agreed to join the study. There were no dropouts (Figure 2).

### 3.3. Feasibility

Table 2 shows the variables related to feasibility. All participants understood the activity during the training session. Only one person (3.3%) had doubts and needed an additional session with the physiotherapist.

All patients completed the intervention and performed a minimum of 42 days of activity (20 repetitions/day).

Regarding perceived load, 100% of the participants considered the exercise adequate. Furthermore, all participants stated that the exercise was not difficult and was easy to do at home without supervision. Regarding the self-perceived effect of the programme, 28 (93.3%) participants considered the intervention beneficial. No one felt that any of the assessed aspects had worsened after the intervention.

Twenty-four (80%) participants encountered no barriers. Six participants encountered difficulties in the implementation of the home programme, the most common being lack of consistency. Facilitators identified included the single exercise in the programme; its simplicity; the time needed to complete it; a video displaying the activity; the possibility of undergoing a treatment at home; and the motivation to improve. Three participants (10%) indicated that they would include a daily reminder to do the activity, one (3.3%) proposed performing it only once a day, and one participant (3.3%) suggested doing it fewer days per week.

### 3.4. Outcome Measurements

All variables were collected at both T0 and T1. At T0, normal distribution was found in all the numerical variables except: elbow ST, external knee ST, internal knee ST, elbow CPM, internal knee CPM, SF12, KOOSSR, and KOOSQL. At T1, only the following variables showed a normal distribution: external knee ST, CSI, SF12, KOOSP, KOOSSP, and KOOSQL. (Supplementary S2) Table 3 shows means, standard deviations, medians, and interquartile ranges for all the variables at T0 and T1.

Student’s *t*-test was carried out for the variables NRS, external knee CPM, CSI, and KOOSP, whereas the Wilcoxon test was used for the rest of the variables. Statistically significant differences (*p* < 0.05) were found in NRS, elbow PTT, external knee PPT, internal knee PPT, elbow CPM, internal knee CPM, CSI, and in all five subscales included in the KOOS. Regarding the effect size for those variables where significant differences were found, large effect sizes were found for NRS, CSI, KOOS symptoms, KOOS pain, and KOOS activities of daily living; and medium effect sizes were found for elbow PPT, external knee PPT, internal knee PPT, elbow CPM, KOOS functioning in sports and recreation, and KOOS quality of life. Table 4 shows the effect sizes for the variables that showed significant differences between T1 and T0. For variables that have shown a normal distribution, mean differences (standard deviation), confidence intervals, and Cohen’s d are included. For variables that did not show a normal distribution, the z-statistic of the Wilcoxon test, the total number of observations, and the r are included.

No adverse effects were reported in any of the participants.

## 4. Discussion

This is the first research to evaluate the feasibility and effects of a home-based neural self-mobilisation programme in participants with KO. Feasibility studies demonstrate the relevance, sustainability, and appropriateness of interventions for further clinical trials. The success of clinical studies depends on user participation and satisfaction, a crucial factor that allows researchers to design and optimise interventions [38]. This research revealed that a self-administrating neurodynamic program at home was feasible and safe. Participants also perceived that it was a simple intervention to do at home without the need for additional equipment or supervision. They also reported improvements in knee pain, mobility, and swelling.

The study included 30 patients, most of whom were women. It should be noted that KO in people over the age of 60 affects more women than men [39]. The mean BMI was 25.88 (±4.1), which is classed as overweight.

### 4.1. Feasibility

The self-administered, home-based neurodynamic programme resulted in high participation. This may be because KO is a chronic degenerative disease and therefore these patients require long-term treatment for their symptoms [11].

In this study, 100% adherence was found. Previous studies of telerehabilitation in patients with KO have shown adherence rates of between 90% and 100% [24,25,26], which is quite similar to our results.

One hundred percent of participants completed a minimum of 42 days of intervention over 8 weeks. Participants were encouraged to complete the maximum number of 56 sessions, and 14 (46.7%) of them did so. Twenty-two (73.3%) participants completed the morning sessions and afternoon sessions, while the rest completed all the repetitions of the day in a row. The main reason given was that their work and family life made it difficult for them to do the morning and afternoon repetitions.

All participants reported that it took them less than 10 min/day to complete the activity, which facilitated programme follow-up, as they would not have enough time for other, longer interventions. The importance of having the necessary time to carry out the therapy has already been pointed out [40], as well as the fact that telerehabilitation allows the patient to choose the time to carry out the interventions, allowing the therapies to be adapted to each patient’s daily life [26]. In addition, most of the participants had a fixed schedule for the home programme, which has been shown to increase adherence to treatment through habit formation [41].

All participants understood the exercise in the training session; only one (3%) of them had doubts, which required a supervised session with the physiotherapist. The simplicity of the exercise and the availability of a video showing the activity made it easy to understand. This is an advantage for patients with this chronic disease, as it reduces treatment costs by requiring fewer supervised sessions.

All participants found the programme suitable for them and easy to do at home, without professional support. This, together with the fact that no equipment or modifications to daily activities are required, makes it a low-cost exercise suitable for KO patients. As KO is a chronic disease that requires long-term treatment with high economic costs [2,3], this home-based programme could be a good alternative or complementary treatment. Moreover, since patients do not require any assistance to perform the exercise, it increases the autonomy and empowerment of KO patients in their self-care [42]. The intervention showed positive self-perceived effects on pain, function, and swelling. No adverse effects were found, which could indicate that this treatment is suitable for the KO population. The elderly nature of people with KO should be considered, which may mean that other, more aggressive therapies may interact with drug treatments and joint replacement [43,44].

Eighty per cent of the participants reported no barriers to the intervention. Few participants who identified barriers said they found it difficult to do the activity twice a day or that they could not do any sessions due to family issues. These barriers can be avoided, either by modifying the programme or by increasing participants’ motivation or follow-ups. Regarding facilitators, the simplicity and duration of the programme, as well as the fact that only one exercise was included, and the availability of a video were highlighted. Previously, the main barriers to physical activity for patients with KO were pain, fatigue, and stiffness [45], and our intervention helped patients to reduce them. Another barrier reported previously was social comparison [45]. This was not the case in our programme, which is carried out individually. The last barrier was the cost [45], but our program does not require any additional material. The literature indicates reduction in pain and stiffness, increased function, and support from physiotherapists as facilitators [45]. In our program, participants were able to have contact with a physiotherapist.

### 4.2. Outcome Measurements

All data were collected at both T0 and T1. Statistically significant improvements were found in PPT and elbow CPM with medium effect sizes. No previous studies were found to evaluate the effect of neural mobilisation on pain in KO. Courtney et al. [46] found that joint mobilisation improved PPT and CPM in KO patients, with medium effect sizes.

We also found a statistical improvement in perceived pain intensity (NRS), with a large effect size (d = 1.61). This is the first research to evaluate this. Alghadir et al. [47] found statistical effects on NRS after exercise therapy with a large effect size, while Bhagat et al. [48] reported improvements after the use of Mulligan mobilisations.

For the KOOS, we found statistically significant differences with large and medium effect sizes. Previously, exercise programmes in KO patients achieved statistically significant results on the KOOS questionnaire with medium effect sizes [49,50]. Statistically significant results were also found for the CSI questionnaire, with a large effect size (d = 0.92). Lluch et al. [32], using education and mobilisation, found a moderate effect size for the CSI questionnaire. No changes in TS were found, suggesting that passive oscillating mobilisation may be a preferable option for activating the descending pain chain compared to other active therapies in KO patients [46,51].

The goal of this research was to assess the feasibility of a home-based neurodynamic programme for people with KO. As stated above, participants were over 50 years of age, most were women (70%), and the mean BMI was 25.88 (±4.1). KO is most frequent in elderly people, as age is a risk factor [1,4,5]. Furthermore, the prevalence is higher in women than in men [39]. The participants in our study, therefore, meet the most common characteristics in terms of age, sex, and BMI of people suffering from KO. There is evidence that a BMI greater than 25—comprising both overweight and obesity—is a risk factor for KO [52]. An increase in BMI above 25 can lead to reduced functionality, so simple exercise programmes, such as the one used in this research, may be an appropriate therapy to reduce symptoms and improve functionality in people with KO.

A clinical study was conducted to assess the reliability of a home-based neurodynamic programme. These studies are costly and time-consuming, and require participants to be followed up with over time. For this reason, it could be interesting in the future to include computational simulation studies, which reduce costs and provide results more quickly [53,54,55]. Computational prediction models have been designed for total knee arthroplasty to evaluate biomechanical effects [56] and patient-reported outcomes, including KOOS [57]. This simulation decreases the likelihood of impairments after total knee arthroplasty and may be useful in planning [58]. These models provide a better understanding of the likely clinical outcomes of an intervention than would exist without their use and have the potential to help in choosing the best treatment [59].

### 4.3. Limitations

The present study is part of a larger study, so it should be noted that in this feasibility research, the follow-up of participants was short-term, and measurements were only collected before the start and after the end of the home-based programme.

In addition, the sample size of the present study was small, so the results should be interpreted with caution. The main study should be conducted with an adequate sample size. In addition, participants should be followed up with over time to see if any positive effects are sustained in the long term.

Therefore, more rigorous studies involving patients with different degrees of KO, BMI, and activity levels, as well as a control group, are needed before this programme can be recommended.

## 5. Conclusions

A home-based, active neurodynamic programme has been shown to be a feasible and safe intervention in patients with grade I–II KO. The use of a single, simple exercise, supplemented by a video showing the activity and weekly follow-ups by a physiotherapist, has been shown to facilitate the adherence to the programme. In addition, this technique has shown positive effects on pain and function.

## Figures and Tables

**Table 1:** Demographic variables: descriptive analysis.

		Frequency (%)
Gender	Male Female	9 (30) 21 (70)
Kellgren–Lawrence scale	Grade I Grade II	6 (20) 24 (80)
Lower limb deformities	Yes No	2 (6.66) 28 (93.33)
Analgesic intake	Yes No	5 (16.66) 25 (83.3)
	Mean (SD)	Median (IQRs)
Age (years)	66.27 (±8.93)	67.5 (72–67.5)
Height (m)	1.64 (±0.08)	1.65 (1.58–1.69)
Weight (kg)	70.05 (±14.78)	67.5 (60.25–79)
BMI (kg/m^2^)	25.88 (±4.1)	25.5 (23.11–27.29)

IQR: interquartile range; SD: standard deviation.

**Table 2:** Variables related to comprehension, adherence, load, and self-perceived effects, barriers, and facilitators: descriptive analysis.

Comprehension		Participants (30)
Understanding of the activity during the training session (*n* (%))	Strongly agree Agree Neutral Disagree Strongly disagree	29 (96.66) 1 (3.33) 0 (0) 0 (0) 0 (0)
Doubts during the intervention period (*n* (%))	Yes No	1 (3.33) 29 (96.66)
Watched video during the intervention period (*n* (%))	Yes No	14 (46.66) 16 (53.33)
Video views	Mean (SD)	0.83 (1.02)
Calls to the researcher (*n* (%))		0 (0)
Additional sessions (*n* (%))	Yes No	1 (3.33) 29 (96.66)
Adherence		
Participation (*n* (%))	Yes No	30 (100) 0 (0)
Participants who completed the programme (*n* (%))	Yes No	30 (100) 0 (0)
Number of days the programme was carried out (*n* (%))	56 days 52 days 51 days 50 days 49 days 46 days	14 (46.66) 8 (26.66) 1 (3.33) 2 (6.66) 4 (13.33) 1 (3.33)
Time of day when the programme was performed (*n* (%))	Morning and afternoon Morning or afternoon only	22 (73.33) 8 (26.66)
Daily time (minutes) to carry out the programme	Mean (SD)	3.5 (±1.22)
Fixed schedule to carry out the programme (*n* (%))	Strongly agree Agree Neutral Disagree Strongly disagree	3 (10) 14 (46.66) 8 (26.66) 3 (10) 2 (6.66)
Load		
It was appropriate (*n* (%))	Strongly agree Agree Neutral Disagree Strongly disagree	21 (70) 9 (30) 0 (0) 0 (0) 0 (0)
It was difficult (*n* (%))	Strongly agree Agree Neutral Disagree Strongly disagree	0 (0) 0 (0) 0 (0) 10 (33.33) 20 (66.66)
The difficulty increased over the days (*n* (%))	Strongly agree Agree Neutral Disagree Strongly disagree	0 (0) 0 (0) 0 (0) 11 (36.66) 19 (63.33)
The difficulty decreased over the days (*n* (%))	Strongly agree Agree Neutral Disagree Strongly disagree	3 (10) 7 (23.33) 11 (36.66) 7 (23.33) 2 (6.66)
Easy to perform at home (*n* (%))	Strongly agree Agree Neutral Disagree Strongly disagree	21 (70) 9 (30) 0 (0) 0 (0) 0 (0)
Difficult to perform without physiotherapist personal supervision	Strongly agree Agree Neutral Disagree Strongly disagree	0 (0) 0 (0) 1 (3,33) 11 (36.66) 18 (60)
Why (*n* (%))	Easy Video Both: video and easy	18 (60) 8 (26.66) 4 (13.33)
The programme was burdensome (*n* (%))	Strongly agree Agree Neutral Disagree Strongly disagree	0 (0) 0 (0) 5 (16.66) 6 (20) 19 (63.33)
It was difficult to combine it with the ADL (*n* (%))	Strongly agree Agree Neutral Disagree Strongly disagree	0 (0) 0 (0) 1 (3.33) 8 (26.66) 21 (70)
It was necessary to modify any ADL to perform the programme (*n* (%))	Strongly agree Agree Neutral Disagree Strongly disagree	0 (0) 0 (0) 0 (0) 7 (23.33) 23 (76.66)
It was necessary to stop doing any ADL to carry out the programme (*n* (%))	Strongly agree Agree Neutral Disagree Strongly disagree	0 (0) 0 (0) 0 (0) 6(20) 24 (80)
I had the required equipment at home (*n* (%))	Strongly agree Agree Neutral Disagree Strongly disagree	28 (93.33) 2 (6.66) 0 (0) 0 (0) 0 (0)
I had to make some home modifications to perform the programme (*n* (%))	Strongly agree Agree Neutral Disagree Strongly disagree	0 (0) 0 (0) 0 (0) 4 (13.33) 26 (86.66)
Someone helped me to carry out the programme (*n* (%))	Strongly agree Agree Neutral Disagree Strongly disagree	0 (0) 0 (0) 0 (0) 2 (6.66) 28 (93.33)
Self-perceived effect		
The programme was good (*n* (%))	Strongly agreeAgreeNeutralDisagreeStrongly disagree	15 (50)13 (43.33)2 (6.66)0 (0)0 (0)
I felt improvements in (*n* (%))	Pain Mobility Pain and mobility Pain, mobility, and swelling Flexibility Flexibility and pain Did not improve	15 (50) 3 (10) 8 (26.66) 1 (3.33) 1 (3.33) 1 (3.33) 1 (3.33)
There were no changes in (*n* (%))	Anything Pain Mobility Swelling	23 (76.66) 1 (3.33) 2 (6.66) 4 (13.33)
Barriers		
Barriers (*n* (%))	Anything Lack of consistency Remembering exercise Family obligations Better to do once a day	24 (80) 3 (10) 1 (3.33) 1 (3.33) 1 (3.3)
Facilitators		
Facilitators (*n* (%))	Simplicity Duration Single exercise Video Motivation to improve Home treatment	23 (76.66) 2 (6.66) 2 (6.66) 1 (3.33) 1 (3.33) 1 (3.33)

ADL: Activities of Daily Living.

**Table 3:** Outcome measurement at T0 and T1: descriptive analysis.

	T0		T1	
	Means (SD)	Medians (IQR)	Means (SD)	Medians (IQR)
NRS	5 (1.90)	5 (3.35–6.18)	1.93 (1.25)	1.75 (.94-.3.2)
Elbow PPT	2.54 (0.77)	2.5 (1.83–3.1)	2.90 (1.03)	2.75 (1.98–3.58)
External knee PPT	2.65 (1.15)	2.58 (1.71–3.38)	3.15 (1.49)	2.58 (2.21–3.98)
Internal knee PPT	2.5 (1.11)	2.38 (1.5–3.21)	3.12 (1.72)	2.5 (1.83–3.94)
Elbow TS	1.48 (0.69)	1.33 (1–1.67)	1.49 (0.46)	1.33 (1.25–1.58)
External knee TS	1.53 (0.54)	1.33 (1.2–1.69)	1.45 (0.35)	1.37 (1.22–1.58)
Internal knee TS	1.55 (0.62)	1.33 (1.23–1.67)	1.64 (0.66)	1.53 (1.26–1.71)
Elbow CPM	1.32 (0.41)	1.24 (1.08–1.35)	1.68 (0.62)	1.5 (1.33–2.08)
External knee CPM	1.38 (0.66)	1.12 (1–1.47)	1.59 (0.56)	1.5 (1.31–2)
Internal knee CPM	1.48 (0.69)	1.27 (1.07–1.67)	1.94 (1.24)	1.67 (1.33–2)
CSI	27.97 (9.76)	28 (20–32)	24.20 (9.26)	23 (16.75–29)
SF12	32.17 (2.21)	32.5 (30–34)	31.90 (2.25)	32 (30.75–33)
KOOSS	59.52 (21.27)	60.71 (42.86–75)	76.43 (14.67)	67.86 (66.96–92-86)
KOOSR	52.13 (15.96)	51.39 (41.69.44)	66.48 (13.51)	68.06 (54.86–75)
KOOSADL	58.48 (14.14)	60.29 (45.59.66.54)	72.35 (15.92)	74.27 (54.41–83.82)
KOOSSP	25.60 (21.71)	22.5 (5–50)	38.63 (23.76)	35 (15–60)
KOOSQL	40.21 (19.26)	30.5 (25–57.81)	50.83 (18.7)	50 (37.5–62.5)

CPM: Conditioned pain modulation; CSI: Central Sensitization Inventory questionnaire; IQR: interquartile range; KOOSADL: Knee Injury and Osteoarthritis Outcome Score Activities of Daily Life; KOOSQL: Knee Injury and Osteoarthritis Outcome Score Quality of Life; KOOSP: Knee Injury and Osteoarthritis Outcome Score Pain; KOOSS: Knee Injury and Osteoarthritis Outcome Score Symptoms; KOOSSR: Knee Injury and Osteoarthritis Outcome Score Sports and Recreations; NRS: numerical rating scale; PPT: pressure pain threshold; SF12: 12-item Short Form Survey questionnaire; TS: Temporal summation; T0: pre-treatment; T1: post-treatment.

**Table 4:** Effect sizes for the variables that showed significant differences between T1 and T0 (*p* < 0.05).

	Means (SD)	CI	Cohen’s d (CI)
NRS	−3.06 (1.91)	−3.78; −2.35	−1.61 (−1.05; −2.14)
external knee PPT	0.51 (0.96)	0.15; 0.87	0.53 (0.91; 0.15)
CSI	−3.77 (4.1)	−5.3; −2.24	-0.92 (−1.34; −0.48)
KOOSP	14.35 (11.11)	10.20; 18.50	1.29 (0.8; 1.77)
-	z	N	r
elbow PPT	−3.097	60	−0.4
internal knee PPT	−3.172	60	−0.41
elbow CPM	−3.028	60	−0.391
internal knee CPM	−2.099	60	−0.271
KOOSS	−4.431	60	−0.572
KOOSADL	−4.705	60	−0.607
KOOSSR	−3.856	60	−0.498
KOOSQL	−3.398	60	−439

CI: confidence interval; CPM: Conditioned pain modulation; CSI: Central Sensitization Inventory questionnaire; KOOSADL: Knee Injury and Osteoarthritis Outcome Score Activities of Daily Life; KOOSQL: Knee Injury and Osteoarthritis Outcome Score Quality of Life; KOOSP: Knee Injury and Osteoarthritis Outcome Score Pain; KOOSS: Knee Injury and Osteoarthritis Outcome Score Symptoms; KOOSSR: Knee Injury and Osteoarthritis Outcome Score Sports and Recreations; NRS: numerical rating scale; PPT: pressure pain threshold; SD: standard deviation; T0: pre-treatment; T1: post-treatment.

## Data Availability

The data presented in this study are available on request from the corresponding author.

## Supplementary Materials

The following supporting information can be downloaded at: https://www.mdpi.com/article/10.3390/jcm12206635/s1. Survey including questions related to understanding the activity, adherence to the intervention, burden, subjective effect, follow-up, barriers, and facilitators. Supplementary S1: Programme feasibility questionnaire. Supplementary S2: Kolmogorov-Smirnov test for the quantitative variables.
