# Supplementary material for: Active Neurodynamics at Home in Patients with Knee Osteoarthritis: A Feasibility Study
Source: J Clin Med. 2023 Oct 20;12(20):6635. doi: 10.3390/jcm12206635 (PMC10607651; doi:10.3390/jcm12206635)
Supplement: Supplementary file 1 [file jcm-12-06635-s001.zip › jcm-2558419-supplementary.pdf]

Supplementary S1

Understanding the activities:

- Did you understand the activities during the training session?

Strongly agree Agree Neutral Disagree Strongly Disagree

- Did you have any questions about the activities while you were doing the exercise programme at home?

Yes No

Which ones?

- Did you need to watch the video?

Yes No

- How many times?

- Did you have to call the researcher to ask any question?

Yes No

- How many times?

- Did you need a supervised session with the researcher?

Yes No

- How many?

Adherence to the programme:

- If you do not want to take part in the study, why did you decide not to take part? *

- Why couldn't you finish the programme? *

- How many days did you do the programme?

- How many days did you carry out the programme in the mornings and afternoons?

- How long (minutes) did it take you to complete the programme every day?

- Did you have a fixed timetable for the programme?

Strongly agree Agree Neutral Disagree Strongly Disagree

Load:

- Was the programme suitable for you?

Strongly agree Agree Neutral Disagree Strongly Disagree

- Was the programme difficult for you?

Strongly agree Agree Neutral Disagree Strongly Disagree

- Did the difficulty increase as the days went by?

Strongly agree Agree Neutral Disagree Strongly Disagree

- Did the difficulty decrease as the days went by?

Strongly agree Agree Neutral Disagree Strongly Disagree

- Was it easy to carry out the programme at home?

Strongly agree Agree Neutral Disagree Strongly Disagree

- Was it difficult to do the programme without the personal supervision of a physiotherapist?

Strongly agree Agree Neutral Disagree Strongly Disagree

- Why?

- Was the programme a burden for you?

Strongly agree Agree Neutral Disagree Strongly Disagree

- If it was a burden, how did it affect you?

- If it was a burden, why?

- Was it difficult to integrate the intervention into your daily activities?

Strongly agree Agree Neutral Disagree Strongly Disagree

- Did you have to modify your daily activities in order to carry out the programme?

Strongly agree Agree Neutral Disagree Strongly Disagree

- Did you have to stop any of your daily activities to carry out the intervention?

Strongly agree Agree Neutral Disagree Strongly Disagree

- Which and how?

- Did you have the necessary equipment at home to carry out the activity?

Strongly agree Agree Neutral Disagree Strongly Disagree

- Did you have to make any home adaptations to do the programme?

Strongly agree Agree Neutral Disagree Strongly Disagree

- Which adaptations?

- Did you need help from anyone to do the intervention?

Strongly agree Agree Neutral Disagree Strongly Disagree

- Who helped you (cohabitant or someone who came to help you)?

Self-perceived effect

- Do you feel the programme has been good for you?

Strongly agree Agree Neutral Disagree Strongly Disagree

- What has improved?

- What has remained the same?

- What has worsened?

Follow up:

- If you did not participate in the follow-up, why not? *

Barriers:

- What was a barrier to carry out the programme?

Facilitator:

- What was a facilitator to carry out the programme?

- What would you include in the programme to improve it?

		Kolmogorov-Smirnov test	p value
**Demographic variables**	**Age**	.102	.2
	**Height**	.088	.2
	**Weight**	.121	.2
	**Body mass index**	.159	.06
**Variables at T0**	**NRS**	.112	.2
	**Elbow PPT**	.119	.2
	**External knee PPT**	.128	.2
	**Internal knee PPT**	.114	.2
	**Elbow ST**	.258	<.001
	**External knee ST**	.232	<.001
	**Internal knee ST**	.227	<.001
	**Elbow CPM**	.252	<.001
	**External knee CPM**	.133	.188
	**Internal knee CPM**	.258	<.001
	**CSI**	.151	.08
	**SF12**	.163	.041
	**KOOSS**	.15	.083
	**KOOSP**	.128	.2
	**KOOSADL**	.2	.708
	**KOOSSP**	.229	<.001
	**KOOSQL**	.164	.038
**Variables at T1**	**NRS**	.136	.164
	**Elbow PPT**	.164	.038
	**External knee PPT**	.252	<.001
	**Internal knee PPT**	.189	.008
	**Elbow ST**	.213	.001
	**External knee ST**	.15	.083
	**Internal knee ST**	.251	<.001
	**Elbow CPM**	.249	<.001
	**External knee CPM**	.18	.14
	**Internal knee CPM**	.347	<.001
	**CSI**	.132	.191
	**SF12**	.154	.066
	**KOOSS**	.254	<.001
	**KOOSP**	.105	.2
	**KOOSADL**	.168	.03
	**KOOSSP**	.116	.2
	**KOOSQL**	.151	.078

Supplementary S2
